# A Novel Peptide-Based Detection of SARS-CoV-2 Antibodies

**DOI:** 10.3390/biomimetics8010089

**Published:** 2023-02-22

**Authors:** Aliye Bulut, Betul Z. Temur, Ceyhun E. Kirimli, Ozgul Gok, Bertan K. Balcioglu, Hasan U. Ozturk, Neval Y. Uyar, Zeynep Kanlidere, Tanil Kocagoz, Ozge Can

**Affiliations:** 1 Department of Biomedical Engineering, Institute of Biomedical Engineering, Bogazici University, Istanbul 34684, Turkey; 2 Department of Medical Biotechnology, Institute of Health Sciences, Acibadem Mehmet Ali Aydinlar University, Istanbul 34752, Turkey; 3 Department of Biomedical Engineering, Faculty of Engineering and Natural Sciences, Acibadem Mehmet Ali Aydinlar University, Istanbul 34752, Turkey; 4 Genetic Engineering and Biotechnology Institute, Marmara Research Center, TUBITAK, Kocaeli 41470, Turkey; 5 Acibadem Labmed Clinical Laboratories, Istanbul 34752, Turkey; 6 Department of Basic Pharmaceutical Sciences, Faculty of Pharmacy, Acibadem Mehmet Ali Aydinlar University, Istanbul 34752, Turkey; 7 Department of Medical Microbiology, School of Medicine, Acibadem Mehmet Ali Aydinlar University, Istanbul 34752, Turkey

**Keywords:** peptide mimetics, SARS-CoV-2, biosensor, quartz crystal microbalance, antibody detection

## Abstract

The need for rapidly developed diagnostic tests has gained significant attention after the recent pandemic. Production of neutralizing antibodies for vaccine development or antibodies to be used in diagnostic tests usually require the usage of recombinant proteins representing the infectious agent. However, peptides that can mimic these recombinant proteins may be rapidly utilized, especially in emergencies such as the recent outbreak. Here, we report two peptides that mimic the receptor binding domain of the spike protein of severe acute respiratory syndrome coronavirus 2 (SARS-CoV-2) and investigate their binding behavior against the corresponding human immunoglobulin G and immunoglobulin M (IgG and IgM) antibodies in a clinical sample using a quartz crystal microbalance (QCM) sensor. These peptides were immobilized on a QCM sensor surface, and their binding behavior was studied against a clinical serum sample that was previously determined to be IgG and IgM-positive. It was determined that designed peptides bind to SARS-CoV-2 antibodies in a clinical sample. These peptides might be useful for the detection of SARS-CoV-2 antibodies using different methods such as enzyme-linked immunosorbent assay (ELISA) or lateral flow assays. A similar platform might prove to be useful for the detection and development of antibodies in other infections.

## 1. Introduction

Infectious virus investigation and detection methods require quantitative techniques such as biosensors along with conventional studies. The important advantages of biosensors are their ease of use and production, capability for synthetic or natural antibody integration and fast response and high sensitivity, specificity and portability. Therefore, biosensors are very suitable to be used for the detection of microorganisms, not only viruses but also bacteria or fungi. Also, biosensors could be used for the detection of other disease markers or living cells because of their advantages [1]. Early diagnosis is crucial to prevent the severe symptoms of many diseases. Coronavirus Disease 2019 (COVID-19) pandemic has taught us that age and immune system factors are very important for the major cases. For example, the elderly population with severe diseases would be seriously symptomatic. Therefore, early detection of COVID-19 infection with a rapid test is important because COVID-19 has a wide range of symptoms from simple to severe [2,3].

Antibody detections usually include immunoglobulin G (IgG) and immunoglobulin M (IgM) antibodies, and these are conducted with serological tests by using biological fluids such as blood plasma or serum. These tests become crucial for vaccine studies since they can be used to investigate the short-term and long-term antibody response, variability of antibodies and antibody level [4]. Severe Acute Respiratory Syndrome Coronavirus 2 (SARS-CoV-2) tests are usually used for the detection of IgG and IgM antibodies which are also specific for spike glycoproteins (S1 and S2 subunit and receptor binding domain) and nucleocapsid proteins. Frequently used test methodologies are enzyme-linked immunosorbent assay (ELISA), immunochromatographic lateral flow assay, neutralization bioassay and specific chemosensors [5,6]. However, these methods might require some labels, such as radioisotopes or fluorophores [7]. Although these methods are efficiently used, they have some limitations, such as high cost, time-consuming implementation and the requirement for trained personnel [7,8]. Thus, there is a need for small, efficient, portable, fast-responding and sensitive virus biosensors. Biosensors are used for virus or virus protein detection by integrating biological sensing molecules. Biosensors can be classified into three categories with respect to prominently used transducers: optic, piezoelectric and electrochemical. Piezoelectric types can monitor the resonance frequencies (*f*) of certain vibration modes, which may shift upon analyte binding. Quartz crystal microbalance (QCM) sensor belongs to this group [7]. QCM is advantageous compared to the electrochemical or optical methods because it does not require any label such as fluorescence or any electrical conducting. In addition, QCM is a rapid, inexpensive and sensitive method that makes it more useful for detection studies [1,7,9]. Moreover, when comparing QCM with other label-free sensors, such as surface plasmon resonance (SPR) which is an optical sensor, QCM might be considered more useful due to its low cost and portability [10,11]. In principle, when the virus binds to the QCM surface, the fundamental *f* decreases due to the mass increase [9]. Thus, QCM is used for virus detection successfully as a label-free biosensor [1,7,8].

Antibody detection tests usually rely on the design and expression of recombinant proteins that act as an antigen [12]. Quick and efficient recombinant protein expression, especially during emergency situations like pandemics, is not feasible due to the requirement of complex and time-consuming further processes such as cell harvesting, cell lysing and purification [13,14]. Synthetic peptides mimicking recombinant proteins, therefore, become more attractive for quick and affordable applications. [15].

In a previous study, characterization of the receptor binding domains of coronavirus in Middle East respiratory syndrome (MERS), severe acute respiratory syndrome (SARS) and SARS-CoV-2 were studied [16]. Two of the highly conserved peptide regions based on the reference study were chosen, and corresponding peptide sequences were synthesized through solid-phase peptide synthesis. In this study, the binding behavior of these selected regions with the SARS-CoV-2 antibodies in a clinical sample was examined by using a QCM biosensor.

## 2. Materials and Methods

### 2.1. Peptide Synthesis

The following two peptides were synthesized using Fmoc-based solid phase peptide synthesis on a Rink Amide resin (CEM, Lot: RA18003) with a loading capacity of 0.70 mmol/g as a solid support. TBT1: TNGVGYQPYRVVVLSFELLHA and TBT2: DEVRQIAPGQTGKIADYNYKLPDDF. Peptide synthesis was performed on a 0.1 mmol scale with Liberty blue^®^ (CEM, Kassel, Hesse, Germany) peptide synthesizer. The Fmoc protecting groups were removed by piperidine (%20 *v*/*v* in N,N-dimethylformamide). The coupling of amino acids (0.2 M) was performed using N,N-disopropylcarbodiimide (DIC) and ethyl-2-cyano-2-(hydroxyimino) acetate (Oxyma, CEM, Lot: 1802117001-052118, Kassel, Hesse, Germany) in 4 min cycle time (1 min for deprotection, 1 min for washing and 2 min for coupling) at 50 °C. For the cleavage of protecting groups, peptides were treated with a 5 mL cleavage cocktail (TFA/H_2_O/TIS, 95/2.5/2.5, *v*/*v*/*v*) for 30 min at 37 °C. The peptide was precipitated using 15 mL chilled ether solution by centrifugation at 3500 rpm for 5 min. This process was repeated three times.

The crude peptides were analyzed using reverse phase HPLC (Agilent Technologies 1260 Infinity, Santa Clara, CA, USA) with C18 AdvanceBio Peptide Plus 2.1 × 150 mm 2.7-µm column (Agilent Technologies, Santa Clara, CA, USA) at 214 nm. Mobile phase A was water containing 0.05% trifluoroacetic acid (TFA), and Mobile phase B was acetonitrile containing 0.025% TFA. Analysis was carried out with a 5–80% B gradient in 30 min at a flow rate of 0.4 mL/min.

Peptides were purified on a semipreparative VariTide RPC 250 × 10 mm ID column (Agilent Technologies, Santa Clara, CA, USA) at 214 nm at a flow rate of 2 mL/min. The purified peptides were lyophilized for further studies. In addition, synthesized peptides were analyzed with liquid chromatography combined with tandem mass spectrometry (LC-MS/MS). Peptides were analyzed by Agilent Technologies 6420 Triple Quad LC-MS/MS system, equipped with C18, 250 mm × 10 mm ID column. 20 µL peptide (1 mg/mL) was injected into the column, and the analysis was done at a flow rate of 0.5 mL/min via an isocratic method of 50% mobile phase B (acetonitrile with 0.05% formic acid), where mobile phase A was ddH_2_O with 0.05% formic acid.

### 2.2. Preparation of Peptide Conjugate via Maleimide-PEG_8_-NHS Linker

TBT1 and TBT2 peptides were conjugated with Maleimide-PEG_8_-NHS (BroadPharm San Diego, CA, USA) linker to be used in the peptide immobilization step. First, the required amount of peptide was calculated based on the full theoretical surface coverage of the 3-mercaptopropyl trimethoxysilane (MPS)-coated QCM surface [17]. Lyophilized peptide was dissolved in 10 mM phosphate buffer saline (PBS, G-Biosciences, St. Louis, MO, USA), and concentration was calculated via a fluorescent microplate assay for the quantitative measurement of the peptides (Pierce Quantitative Fluorescent Peptide Assay, Thermo Scientific, Waltham, MA, USA). Linker stock solution was prepared in dimethylformamide (DMF, Sigma Aldrich, Burlington, MA, USA) and then diluted in 10 mM PBS. Linker was added in 60-fold molar excess to the conjugation solution. Conjugation occurred at room temperature in a shaker incubator for 30 min. After conjugation was finished, conjugate molecules were separated from the non-conjugated peptide and linker molecules by Sephadex-G25 column (Cytiva, Marlborough, MA, USA) purification. During the running process, 30 mL of distilled water was passed through the column, and 14 vials (approximately 2 mL solution in each vial) were collected at the end. Next, each collected vial was tested on the thin-layer chromatography (TLC) plate along with pure peptide and linker samples to determine the vials that included the conjugate molecule. The solvent used in the TLC procedure was a mixture of 50% dichloromethane (CH_2_Cl_2_) and 50% ethyl acetate (EtOAc). The presence and purity of each fraction were confirmed with HPLC using the same procedure as before. Conjugates were collected, lyophilized and dissolved in 10 mM pH 7.4 PBS. Peptide-linker conjugate solutions were stored at −20 °C for further use.

### 2.3. Preparation of Anti-Human IgG and Anti-Human IgM via EDC/NHS-Mediated FRM Conjugation

As a confirmation of COVID-19 antibody binding, anti-human IgG and anti-human IgM were used in the last step of the experiment. To confirm the result with the fluorescent imaging, anti-human IgG and anti-human IgM were conjugated with the fluorescent reporter microspheres (FRMs) using EDC and NHS reagents (Thermo Scientific, Waltham, MA, USA). In this experiment, two bright blue FRMs (Fluoresbrite BB carboxylate microspheres, Polysciences Warrington, PA, USA) with different sizes were used. FRM 6 µm in diameter was used in the anti-human IgM conjugation, and FRM 1 µm in diameter was used in the anti-human IgG conjugation. FRMs were activated to make them suitable for EDC/NHS coupling of anti-human antibodies. Conjugation reagents were used 100-fold in excess to theoretically cover the entire surface of the FRMs. The required amount of EDC, NHS and 50 µL ddH_2_O was mixed, and then 100 µL FRM was added to the solution. The reaction mixture was vortexed for about 30 s and covered with aluminum folio. Next, the solution was incubated at room temperature in a shaker incubator for 60 min. After incubation, it was centrifuged at 1700× *g* for 5 min, and the supernatant was removed. The pellet was dissolved in 500 µL, 10 mM PBS and was mixed with 375 µL (at a concentration of 1 µg/µL) of anti-human secondary antibody (anti-human IgG or anti-human IgM) and wrapped with the aluminum folio. Afterward, the solution was incubated at 4 °C overnight on an orbital shaker. After overnight incubation, the solution was centrifuged at 1700× *g* for 5 min. The supernatant was removed, and the pellet was dissolved in 2 mL PBS (10 mM, pH 7.4) and vortexed for 1 min. FRM/anti-human antibody conjugate was stored at 4 °C for further use.

### 2.4. QCM Surface Modifications

**Figure 1:**
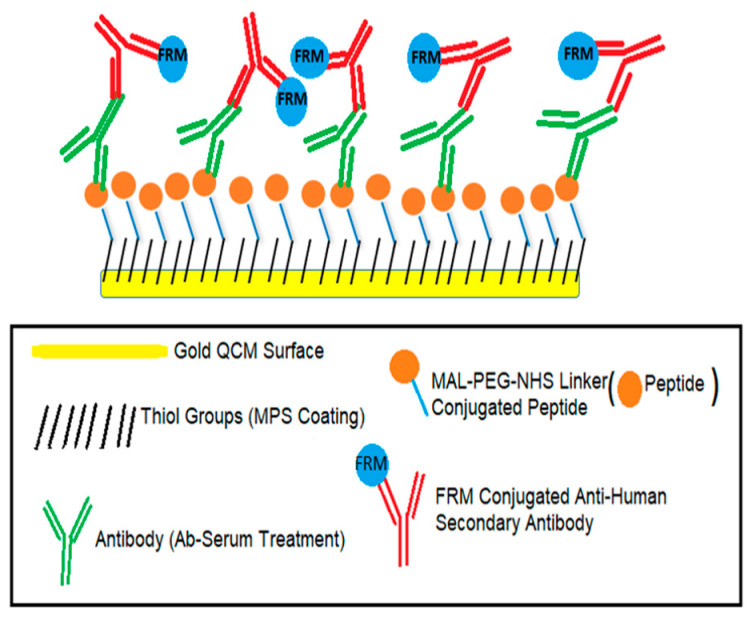
Schematic diagram of the quartz crystal microbalance (QCM) experiment steps. These steps are 3-mercaptopropyltrimethoxysilane (MPS) coating, peptide immobilization, serum treatment (Antibody (Ab) binding) and fluorescent reporter microspheres (FRMs) conjugated anti-human secondary antibody treatment.

Biosensor experiments were conducted in the following order: peptide immobilization, serum treatment, and anti-human secondary antibody treatment. First, the QCM surface was electrically insulated, and then the peptide was immobilized on the surface. Afterward, serum treatment was performed to examine the antibody binding in blood serum. Finally, FRMs conjugated with anti-human secondary antibody treatment were conducted to validate the antibody binding. Peptide immobilization was performed in a flow cell covered with an open cover. Thus, the solution was poured on the surface by pipetting. All the following steps were conducted with the flow setup. The first surface reaction was between the maleimide group of the peptide/Maleimide-PEG_8_-NHS conjugate and the thiol group of the MPS-coated QCM surface. Then, the COVID-19 antibody’s binding with the peptide immobilized on the surface was tested. Finally, FRMs conjugated anti-human secondary antibody (anti-human IgG or anti-human IgM) solution flowed through the surface for the confirmation of antibody binding. The sensor surface experimental setup is illustrated in Figure 1.

### 2.5. Electrical Insulation of QCM Sensor

In this experiment, a 10 MHZ AT-cut QCM sensor was used for the detection purpose, and the surface of the sensor was electrically insulated with MPS (Sigma Aldrich, Burlington, MA, USA) to stabilize the *f* peaks for liquid detection. Before the insulation process, QCM was cleaned in piranha solution (3 parts of 98% sulfuric acid (Sigma Aldrich) and one part of 30% hydrogen peroxide (Sigma Aldrich, Burlington, MA, USA) solution for 1 min. Then, it was rinsed with distilled water and ethanol. Next, QCM was soaked in 0.1 mM MPS solution with 1% distilled water in ethanol for 30 min. Finally, it was soaked in an MPS solution in ethanol consisting of 0.1%MPS and 0.5% ddH_2_O at pH 9.0 for 12 h. This MPS coating was repeated 3 times. Also, QCM was rinsed with distilled water and ethanol between each 12 h coating, and fresh MPS solution was used at each time. To adjust the pH at 9, 11.7 mg potassium hydroxide (Sigma Aldrich, Burlington, MA, USA) was added to the 50 mL MPS solution [18].

### 2.6. Peptide Immobilization

Insulated QCM surface provided free thiol groups, which were used for peptide immobilization. The peptides were conjugated with a MAL-PEG_8_-NHS linker. The linker was conjugated with peptide via the NHS group, and the conjugate molecule provided a free maleimide group on the other side. In the peptide immobilization, when QCM was immersed in the peptide/linker solution, the thiol group of MPS coated sensor surface reacted with the maleimide of the peptide/linker conjugate.

While collecting the QCM *f* data, QCM was placed in a flow cell and covered with an open cover for direct access to the sensor. Then, 200 µL PBS (10 mM) was poured on the surface by pipetting. QCM measurement was started by connecting the flow cell with an impedance analyzer (Keysight E4990A, Santa Rosa, CA, USA). QCM was soaked in the 10 mM PBS solution to reach the stable *f,* and then, 40 µL peptide/linker conjugate solution was poured on the surface by pipetting. QCM was immersed in peptide/linker solution for 90 min, and *f* data were real-time monitored during the experiment. After peptide immobilization, QCM was washed with PBS and then treated with 1% BSA in 10 mM PBS for 1 h to block the nonspecific binding for antibody detection in human blood serum treatment.

### 2.7. Resonance Frequency Measurement and Flow Setup

**Figure 2:**
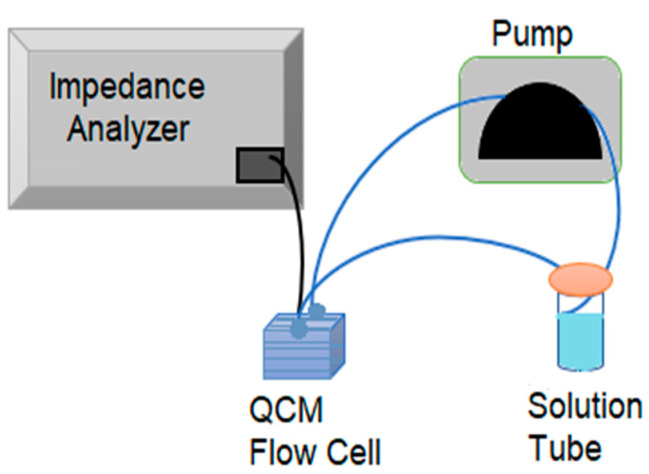
Schematic diagram of the QCM experiment flow setup.

As mentioned previously, the resonance frequency of the QCM sensor was measured by using a Keysight E4990A impedance analyzer. A “moving window” algorithm mentioned in the reference [10] was used to collect the data and monitor the *f* in real time until the recording was stopped by the experimenter. All *f* data, except peptide immobilization, were collected with flow setup. The flow setup consists of a flow cell (openQCM sensor module/openQCM-SENS-MOD-03, Pompeii, NA, Italy), a solution tube, a tubing system and a peristaltic pump (Masterflex L/S 7534-04, Wayne, PA, USA), as shown in Figure 2. The QCM sensor was placed in the flow cell and covered with a fluidic cover consisting of a window. The flow cell was connected to the solution tube with tubing, and the flow was carried out by a peristaltic pump at a 0.67 mL/min flow rate for all experiment steps. After serum treatment was finished, the tubings were replaced with PBS solution. In PBS wash, inlet and outlet tubings were placed in separate containers for 10 min to prevent recirculation. Afterward, FRM/anti-human secondary antibody solution tube was connected, as shown in Figure 2. BSA blocking, serum and FRM/anti-human secondary antibody treatments were all carried out with a flow setup. In addition, PBS wash was performed for 10 min between each detection step. The concentration of PBS solution used for all QCM experiments was 10 mM.

QCM detections normally follow a 3-σ limit of detection principle, where a shift in the resonance frequency would be considered a detection as long as the amplitude of the shift is 3 times or more than the -baseline- standard deviation (SD) of the resonance frequency in the respective control experiment [19].

### 2.8. Detection of COVID-19 Antibody Binding

COVID-19 PCR-negative/antibody-positive and PCR-negative/antibody-negative blood serums of two participants were used in the study. Participants were asymptomatic but had a risk of COVID-19; therefore, they applied at Acibadem Altunizade Hospital to have COVID-19 PCR and antibody testing. All subjects gave their informed consent for inclusion before they participated in the study. Ethical approval for the study was obtained from Acibadem University Ethical Committee, ATADEK, with approval No: ATADEK-2022/12.

All the cases were confirmed by RT-PCR (Bio-speedy^®^ Direct RT-qPCR SARS-CoV-2 Bioeksen, Istanbul, Turkey) at RT-PCR instrument (Biorad CFX96, Hercules, CA, USA) of nasopharyngeal and oropharyngeal swabs (vNAT^®^ Transphere Tube, Bioeksen, Istanbul, Turkey). Antibody detection was performed with multiple kits (Siemens, Erlangen, Germany and Roche, Basel, Switzerland, Chemiluminescence Immunoassay (CLIA); Euroimmun, Waltham, MA, USA and Aesku, Wendelsheim, Germany, ELISA). Both CLIA kits were used to examine the total antibody, and both ELISA kits were used for the detection of IgG. IgM was tested with an Aesku ELISA kit.

Antibody binding detection was performed to examine the interaction between the synthesized peptide and the COVID-19 antibody. Analyses were carried out in human blood serum, followed by BSA blocking. QCM was washed with PBS, and then serum solution was sent through the flow cell. The same PCR negative/antibody positive serum and the PCR negative/antibody negative serum were used for all detection and control experiments, respectively. 20 µL serum was diluted in 3 mL PBS and detection was carried out for at least 1 h. Serum treatment was examined for both TBT1 and TBT2 peptides.

### 2.9. FRM Conjugated Anti-Human Secondary Antibody Confirmation for Antibody Binding Detection

Anti-human secondary antibody detection was performed after PBS wash following the antibody binding. FRMs conjugated anti-human secondary antibody was used to examine the QCM *f* change and visualize the surface for validation of antibody binding. 25 µL FRM/anti-human secondary antibody conjugate solution was diluted in 3 mL PBS and flowed through the sensor while measuring the *f* during the experiment. The solution tube was covered with aluminum folio during the experiment to protect the FRMs from light. In this step, anti-human IgM conjugated with FRMs of 6 µm diameter was used only in the TBT1 experiments, and anti-human IgG conjugated with FRMs of 1 µm diameter was used in both TBT1 and TBT2 experiments.

### 2.10. Fluorescence Microscopy Confirmation of QCM Detection via FRM

Bright blue, fluorescent reporter microspheres were used as the visualization agent. After all the experiments were finished, QCM was separated from the flow cell and examined under the fluorescence microscope (Zeiss Axio Vert.A1, Oberkochen, Germany). DAPI filter was used for imaging. FRM particles were counted using the DotCount software provided by the Laboratory for Computational Longitudinal Neuroimaging (LCLN), Massachusetts Institute of Technology (http://reuter.mit.edu/software/dotcount/, accessed on 1 March 2022). In the software, the image was converted to black-white, and the intensity was adjusted. Then, minimum and maximum dot sizes to be counted were arranged, and FRMs were counted automatically by the software.

## 3. Results

### 3.1. Conjugation of MAL-PEG_8_-NHS to Peptide

**Figure 3:**
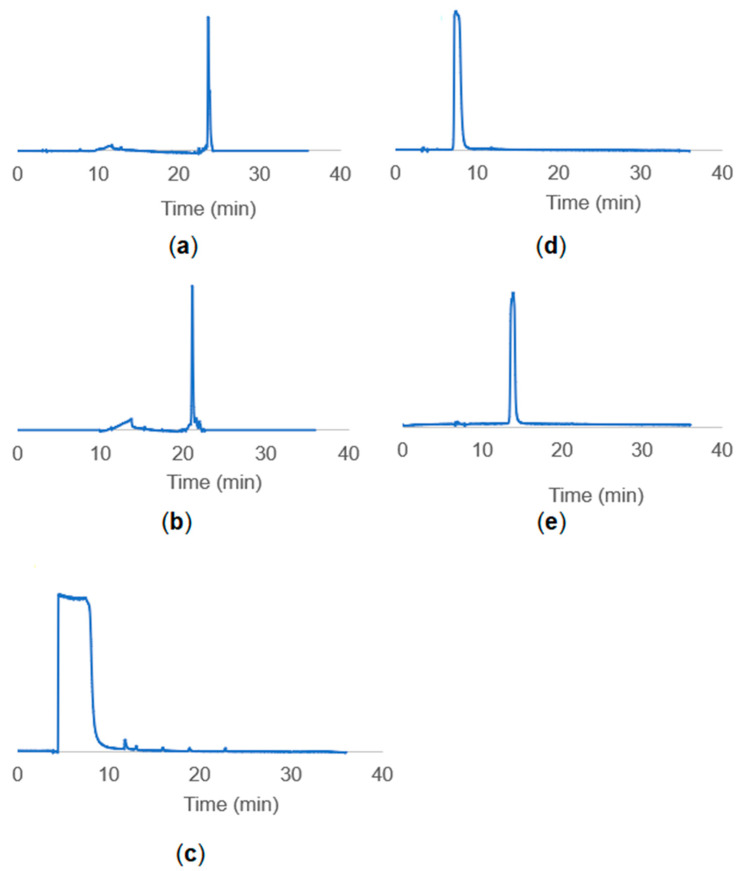
HPLC results of (**a**) pure TBT1 peptide, (**b**) pure TBT2 peptide and (**c**) MAL-PEG_8_-NHS linker molecules and HPLC results of the (**d**) TBT1 conjugates and (**e**) TBT2 conjugates collected after Sephadex-G25 column purification. Scanning was performed for 30 min between 5–80% B gradient at a flow rate of 0.4 mL/min.

Peptide/linker conjugate was purified using a Sephadex-G25 column and analyzed with TLC. Then, HPLC analyses were performed to confirm the purity of the samples. Retention times of the HPLC for pure TBT1 and pure TBT2 peptides were around 23 min and 21 min, respectively. Linker was scanned at 193 nm, and the retention time was between 4–7 min. Figure 3 shows the HPLC results for the pure TBT1, pure TBT2, linker molecules, TBT1 conjugate and TBT2 conjugate collected after Sephadex-G25 column purification.

Also, TBT1 and TBT2 peptides were analyzed with LC-MS/MS for their chemical structures and molecular weight (Figure S1). The TBT1 peptide with a molecular weight of 2363 gmol^−1^ was found to appear in +2, +3 and +5 charges with the corresponding *m*/*z* values as 1192.9, 787.8 and 514.5, respectively. Likewise, the TBT2 peptide’s molecular weight (2867 gmol^−1^) was confirmed by the peaks appearing at 951.6 and 713.8 as *m*/*z* values for its +3 and +4 charged ionization states.

### 3.2. Peptide Immobilization

**Figure 4:**
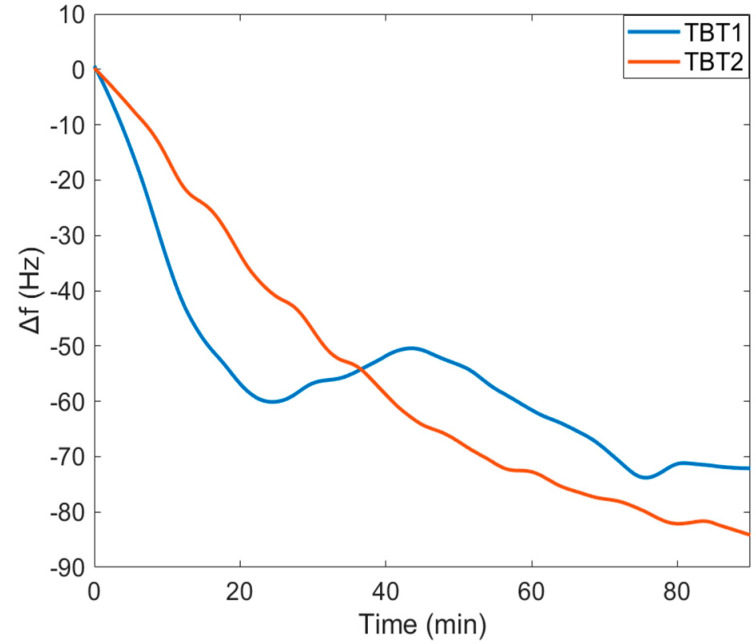
Resonance frequency shifts (∆*f*) observed during TBT1 and TBT2 peptide immobilization on the MPS−coated QCM surface.

Resonance frequency shifts (∆*f*) for both TBT1 and TBT2 during 90 min immobilization are shown in Figure 4. The plot was started at the time when peptide solution was poured on the sensor surface. Two repeated experiments were averaged and shown as one plot for both TBT1 and TBT2 immobilization. ∆*f* values are 71.9 Hz and 83 Hz for TBT1 and TBT2, respectively.

### 3.3. Antibody Binding Detection in Blood Serum

Serum samples used in the study were tested for COVID-19 antibodies via multiple kits and determined as antibody positive or negative. CLIA results for the total antibody of antibody-positive serum were 6.5 (negative < 1) and 44.6 (negative < 1) for Siemens and Roche kits, respectively. ELISA results for IgG of antibody-positive serum were 6.4 and 11.2 for Euroimmun and Aesku kits, respectively. Also, the ELISA result for IgM was 17.8 for the Aesku kit. Control serum was resulted as negative for all antibody tests.

**Figure 5:**
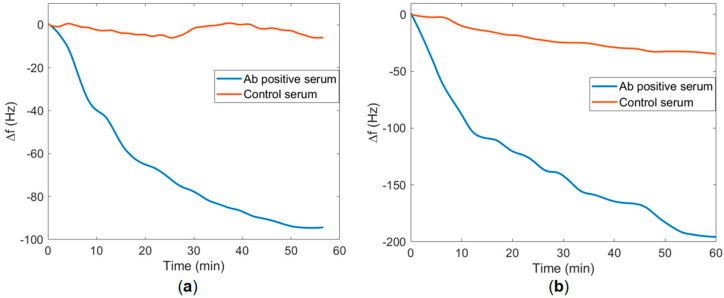
∆*f* versus time of COVID−19 Ab detection by human blood serum treatment for (**a**) TBT1 and (**b**) TBT2.

Interaction between the synthesized peptides and the COVID-19 antibody was examined with the QCM detection experiments. To investigate the binding behavior of peptides and antibodies, antibody-positive blood serum was used for all detections. Also, antibody-negative blood serum was used for all control experiments. The results of ∆*f* observed during serum treatments for TBT1 and TBT2 were shown in Figure 5a and Figure 5b, respectively. TBT1 plot (Figure 5a) shows the average of two repeated experiments of antibody-positive and control serums. The ∆*f* value of TBT1 is 102.5 Hz, and the SD of TBT1 is 0.73 Hz for the antibody-positive and control sera, respectively. In addition, the TBT2 plot (Figure 5b) shows one experiment result of the antibody-positive serum and the average of two repeated experiments of control serum. ∆*f* value of TBT2 is 220 Hz for the positive serum, and the SD is 0.4 Hz for the control serum.

**Figure 6:**
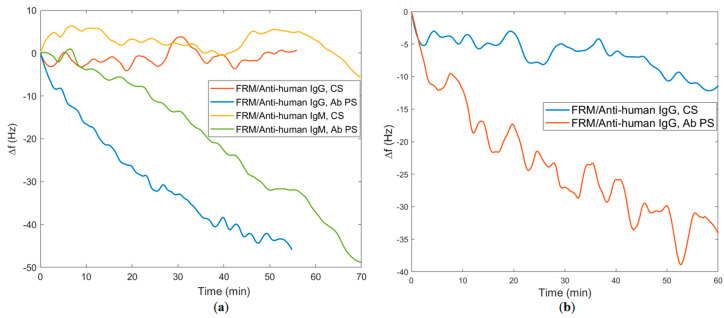
Resonance frequency shifts observed during confirmation experiments for (**a**) TBT1 and (**b**) TBT2. ∆*f* versus time of anti−human immunoglobulin G (anti−human IgG) and FRMs conjugated anti−human immunoglobulin M (anti−human IgM) detections for antibody-positive serum (Ab PS)−treated and negative/control serum (CS)−treated TBT1 are shown in a. ∆*f* of FRMs conjugated anti−human IgG detection for Ab PS−treated and CS−treated TBT2 are shown in (**b**).

After antibody binding, FRMs conjugated with anti-human secondary antibodies were used to confirm the QCM result of antibody binding in blood serum. Results of *f* observed during all these experiments are shown in Figure 6a,b for TBT1 and TBT2, respectively. As seen in Figure 6a, for TBT1, ∆*f* values of anti-human IgG and FRMs conjugated anti-human IgM detections are 48 Hz and 41.8 Hz for antibody-positive serums, and the SD values are 1.7 Hz and 2.55 Hz for control serums. In addition, as seen in Figure 6b, for TBT2, the ∆*f* value of FRMs conjugated anti-human IgG detection is 33 Hz for antibody-positive serum, and the SD is 2.56 for control serum. Resonance frequency shifts (Hz) of TBT1 and TBT2 immobilizations, positive serum antibody detections and anti-human IgG and anti-human IgM conjugated FRM confirmations were tabulated in Table 1 for a clearer overview.

### 3.4. Fluorescence Microscopy Confirmation of Antibody Binding via FRM

**Figure 7:**
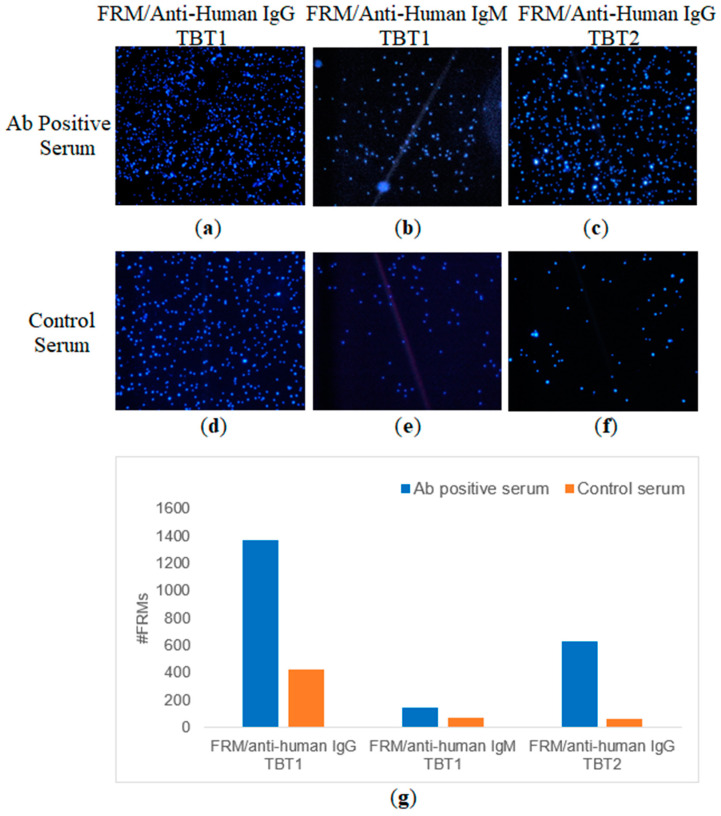
Blue, fluorescent images of the captured microspheres on the QCM surface after FRMs conjugated secondary antibody detection following Ab positive (**a**–**c**) and control serum (**d**–**f**) treatments. (**a**,**d**) are the results of FRM/anti-human IgG treatments, (**b**,**e**) are the results of FRM/anti-human IgM treatments for TBT1. Also, (**c**,**f**) are the results of FRM/anti-human IgG treatments for TBT2. (**g**) shows the number of FRMs captured on the QCM surface after various treatments for TBT1 and TBT2. The numbers of the FRMs captured on the QCM surface are 1368, 140, 631, 419, 72 and 58, which are shown in (**a**–**f**), respectively.

Antibody binding with peptide was confirmed by fluorescent microscopy at the end of QCM experiments. Optical micrographs of FRMs captured on the QCM surfaces were visualized under the fluorescent microscope, as shown in Figure 7a–f. The FRMs used in the experiments for anti-human IgG and anti-human IgM conjugations were 1 µm and 6 µm in diameter, respectively.

## 4. Discussion

The recent COVID-19 pandemic taught us that early diagnosis and rapidly developed detection techniques are important to fight against the outbreak. Antibody tests are important for investigating the immunity of a population [12]. In this study, the antibody test was studied by using synthesized biomimetic peptides. HPLC results of TBT1 (Figure 3a) and TBT2 (Figure 3b) reveal that the purity of the peptides was 99%. Figure 3d,e indicate the conjugation of purified PEG linker (Figure 3c) with TBT1 and TBT2 peptides, respectively. As seen in Figure 3d,e, since the constructs became more hydrophilic after conjugation, corresponding peaks have shifted to the left. Therefore, conjugation reactions were performed with high efficiency.

When TBT1 and TBT2 structures were estimated using PEPFOLD [20,21,22], it seems like these peptides form a near-alpha-helical structure. Using the diameter of an alpha helix and the total available area of the sensor surface, we were able to estimate the amount of peptide that would be needed to cover the surface. Results of ∆*f* for both TBT1 and TBT2 peptide immobilizations indicate that there was a significant decrease in *f* for both TBT1 and TBT2 immobilization, and the surface became saturated within 90 min (Figure 4). In addition, when comparing with the control serum sample, Figure 5 shows a significant ∆*f* resulting from the binding between COVID-19 antibody from the positive serum sample and peptide for both TBT1 (Figure 5a) and TBT2 (Figure 5b). Antibody-positive serum experiments resulted in approximately 100 Hz and 200 Hz ∆*f* for TBT1 and TBT2, respectively. This might be due to several reasons, such as the higher binding affinity of TBT2, different binding kinetics of TBT2 from TBT1, or steric hindrance of TBT1 as well as viscoelastic differences upon binding of different peptides. TBT1 binds to the surface via the only amine group of the N-terminal, and the lower reactivity of TBT1 might be due to the steric hindrance, which might affect the antibody binding. In addition, TBT2 binds to the surface through both the N-terminal and two other lysines in its structure, and this makes TBT2 to be able to bind to the surface in different orientations. Therefore, the surface orientation of TBT2 might affect the viscoelastic differences on the surface, providing more flexibility to bind to the antibody.

QCM that was used in this study was first used in the gas phase, and it is known that viscosity and the density of solution have an effect on the QCM signal in liquid detection [23,24]. Therefore, QCM measurements were not aimed at examining the concentration dependency, and ∆*f* indicates the detection qualitatively. One parameter affecting the *f* is penetration depth. As mentioned previously, TBT2 may bind to the surface sidewise and in other directions, and it might change the sensing depth due to the viscoelastic changes which may rise from this orientation change.

Since individual and total antibody detections were performed with multiple commercially available kits, results can be considered reliable. Peptide and antibody binding interactions were examined with QCM by using previously tested antibody-positive and control serums. Confirmation of antibody binding by using anti-human secondary antibodies was also examined with both QCM detection and FRM imaging studies (Figure 6 and Figure 7). Both IgG and IgM binding for TBT1 were validated with ∆*f,* shown in Figure 6a, and the FRM images, shown in Figure 7. Anti-human secondary antibody (anti-human IgG or anti-human IgM) binding resulted in the accumulation of FRMs on the surface and the detection of ∆*f*, which confirm not only the presence of COVID-19 antibody but also the interaction of antibody with TBT1 immobilized on the surface. FRMs have a significant number of carboxylic acid active groups on their surface, most of which are active even after IgG or IgM conjugation. Because of the significant size difference (IgM is approximately 6 times as big as IgG) between IgG and IgM, the non-specific binding of these active groups on TBT1 might have been hindered in the case of IgM binding. This would explain why there is less IgM binding in the control serum compared to IgG binding in the control serum. We also speculate that these active sites on the FRM do not nonspecifically bind to TBT2 as much, because of the sequence and structural differences, even with a higher immobilization efficiency.

## 5. Conclusions

In conclusion, this study showed that synthesized peptides could be utilized for the detection of antibodies against SARS-CoV-2. Developing a practical antibody test by using biomimetic peptides might provide significant advantages in terms of speed and cost of production. Further in-depth studies are needed to validate these results with multiple clinical samples. Peptides used in this study can be incorporated into lateral flow rapid antibody detection tests. The potential of these peptides in vaccine development studies may also be evaluated due to the rapid, simple and easy synthesis and purification steps. Moreover, this platform can be useful for the detection of antibodies against other infections using gold-standard methods such as lateral flow, ELISA or similar assays.

## Figures and Tables

**Table 1:** Resonance frequency shifts (Hz) of TBT1 and TBT2 immobilizations, positive serum antibody detections and anti-human IgG and anti-human IgM conjugated FRM confirmations.

	Immobilization	Positive Serum	FRM Conjugated Secondary Antibody	
			IgG	IgM
TBT1	71.9	102.5	48	41.8
TBT2	83	220	33	NA

## Data Availability

Not applicable.

## Supplementary Materials

The following supporting information can be downloaded at: https://www.mdpi.com/article/10.3390/biomimetics8010089/s1, Figure S1: LC-MS/MS results of TBT1 and TBT2 peptides.
