# Supplementary material for: A Novel Peptide-Based Detection of SARS-CoV-2 Antibodies
Source: Biomimetics (Basel). 2023 Feb 22;8(1):89. doi: 10.3390/biomimetics8010089 (PMC10046560; doi:10.3390/biomimetics8010089)
Supplement: Supplementary file 1 [file biomimetics-08-00089-s001.zip › biomimetics-2195448-supplementary.pdf]

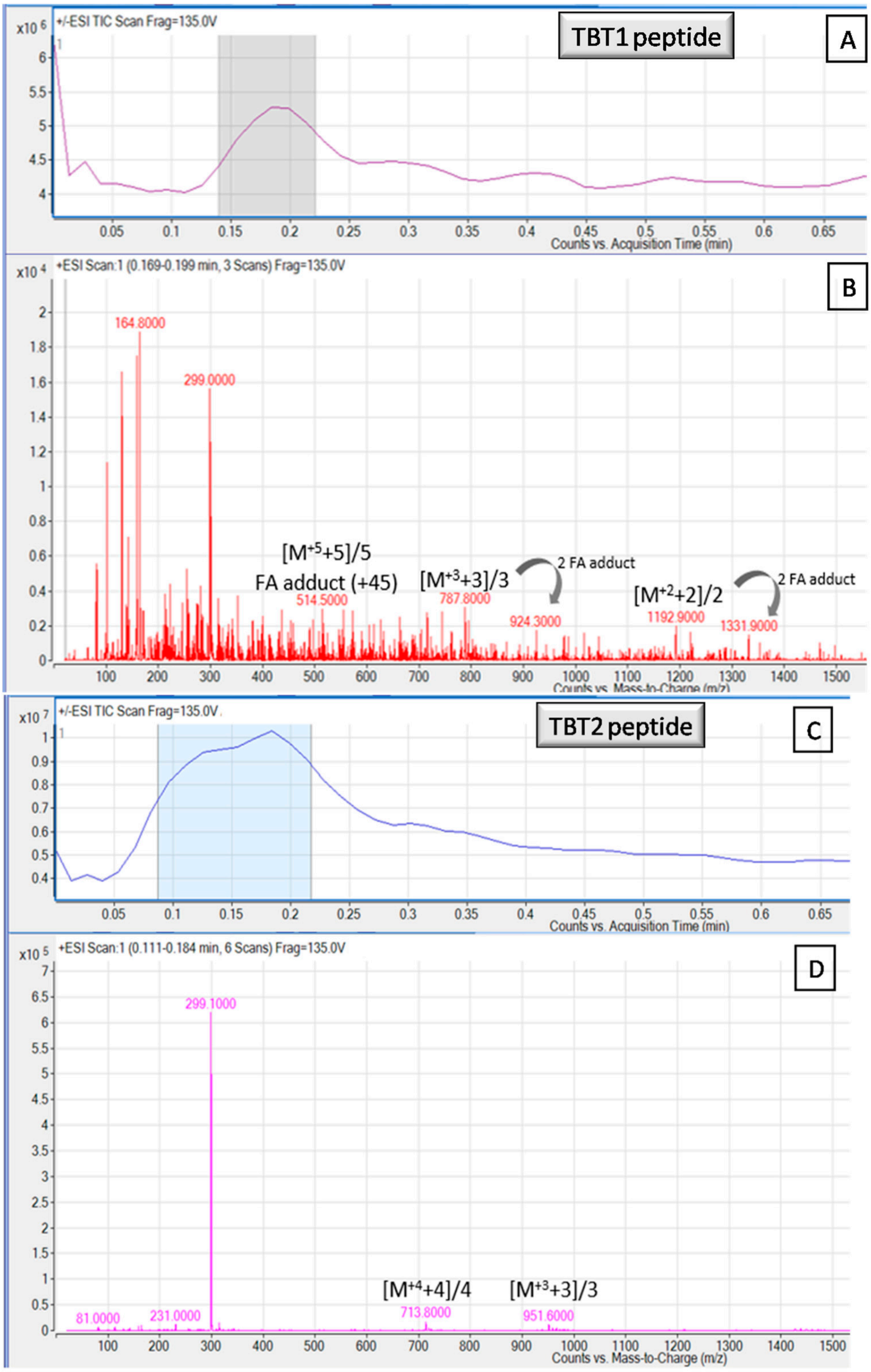


**Figure S1.** LC-MS/MS spectra of (A, B) TBT1 and (C, D) TBT2 peptides. TBT1 peptide with a molecular weight of 2363 gmol$^{-1}$ was found to appear in +2, +3 and +5 charges with the corresponding m/z values as 1192.9, 787.8 and 514.5, respectively (B). Having relatively less intensity, these peaks also appear as their formic acid (FA) adducts, which means that charged peptides are able to make complexes with FA molecules in the mobile phase. TBT2 peptide molecular weight (2867 gmol$^{-1}$) was confirmed by the peaks appearing at 951.6 and 713.8 as m/z values for its +3 and +4 charged ionization states (D).
